# A protocol for enumeration of aquatic viruses by epifluorescence microscopy using Anodisc™ 13 membranes

**DOI:** 10.1186/1471-2180-11-168

**Published:** 2011-07-25

**Authors:** Charles R Budinoff, Star N Loar, Gary R LeCleir, Steven W Wilhelm, Alison Buchan

**Affiliations:** 1 Department of Microbiology, University of Tennessee, Knoxville, Tennessee 37996, USA

## Abstract

**Background:**

Epifluorescence microscopy is a common method used to enumerate virus-like particles (VLP) from environmental samples and relies on the use of filter membranes with pore sizes < 0.02 μm; the most commonly used protocols employ 25 mm Anodisc™ membranes with a built-in support ring. Other filters with small pore sizes exist, including the 13 mm Anodisc™ membranes without a support ring. However, the use of these membranes for viral enumeration has not been previously reported.

**Results:**

Here we describe a modified protocol for 13 mm Anodisc membranes that uses a custom filter holder that can be readily constructed in individual investigators' laboratories from commercially available Swinnex^® ^filter holders. We compared VLP concentrations obtained from phage lysates and seawater samples using both Anodisc membranes, as well as Nuclepore™ small pore-size membranes (0.015 or 0.030 μm). The 13 mm Anodisc membranes gave comparable estimates of VLP abundance to those obtained with the 25 mm Anodisc membranes when similar staining methods were employed. Both Nuclepore membranes typically gave an order of magnitude lower VLP abundance values for environmental samples.

**Conclusions:**

The 13 mm Anodisc membranes are less costly and require smaller sample volumes than their 25 mm counterpart making them ideal for large-scale studies and sample replication. This method increases the options of reliable approaches available for quantifying VLP from environmental samples.

## Background

Viruses are an important component of aquatic food webs. They contribute significantly to the mortality of marine microorganisms and consequently alter species composition and influence the flow of carbon and energy within an ecosystem [1]. As such, accurate and reproducible estimates of virus abundance from environmental samples are essential to our understanding of aquatic biology and biogeochemistry. The earliest estimates of virus-like particles (VLP) in aquatic samples relied on transmission electron microscopy (TEM) [2,3]. However, the high cost, limited availability, and laborious nature of TEM quickly led investigators to switch to epifluorescence microscopy approaches [4-6] using Nuclepore™ track-etched polycarbonate membranes (pore sizes 0.015 or 0.030 μm, Whatman North America) [4,5,7] and methods originally described for enumerating bacteria [8]. Due to slow flow rates, Nuclepore membranes were subsequently replaced by Anodisc™ inorganic (Al_2_O_3_) membranes (pore size 0.02 μm, Anodisc™, Whatman) (refer to Table 1) [9,10]. Anodisc membranes are available in 13 and 25 mm diameters. The 25 mm membrane with a built-in support ring is commonly used to determine VLP abundances in natural systems and is recommended in several published protocols [11,12]. However, the establishment of a protocol using the 13 mm membranes, lacking a support ring, has the advantages of significantly reducing processing costs (by 50% or more; Table 1) and the amount of sample required.

**Table 1:** Specifications of Whatman membranes used in this study

Filter name	Part Number	Filterable Diameter (mm)	Pore Size (μm)	Flow rate^a^	Porosity (pores/cm^2^)	Burst strength (psi)	Autoclavable	Cost per filter (USD)
Anodisc™ 13	6809-7003	13	0.02	4.9, 0.3	10^10^	65-110	yes	2.08
Anodisc 25	6809-6002	21	0.02	4.9, 0.3	10^10^	65-110	No	5.10
Nuclepore™ 15	110601	25	0.015	N/A, 0.002-0.04	10^8^	> 15	Yes	1.84
Nuclepore 30	110602	25	0.03	N/A, 0.06-0.20	10^8^	> 15	yes	1.32

Information obtained from Whatman North America.

^a ^water, air L/min/cm^2 ^@ 10 psi, 25°C.

## Results and Discussion

A practical limitation of the 13 mm Anodisc membranes is the lack of a peripheral support ring to facilitate handling of the membranes. To alleviate this limitation, we constructed custom filter holders and used modifications of traditional protocols for enumeration of VLP. The feasibility of using Nuclepore filters for viral enumerations was also revisited using modified protocols to reduce filtration times. In part, our motivation to reevaluate the feasibility of Nuclepore membranes for VLP enumeration was prompted by production problems of Anodisc membranes [13], which have been subsequently resolved but serve as a reminder that the availability of alternate protocols would be useful.

### Construction of custom filter holders for 13 mm Anodisc membranes

**Figure 1:**
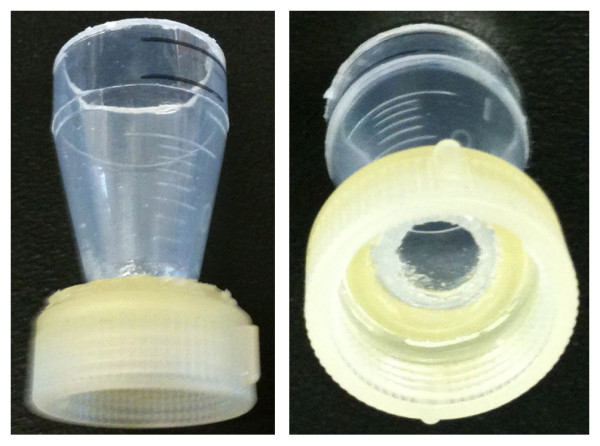
**Custom-built 13 mm filter funnel**. Funnel was assembled from a Swinnex^® ^inlet bonded to the conical end of a 15 ml polypropylene tube.

Filter towers were constructed using the inlet portion of a 13 mm Swinnex filter holder (Millipore, Billerica, MA) that was bonded to a makeshift funnel, the conical end of a 15 mL disposable centrifuge tube (Figure 1). The funnel was necessary as the inlet portion could only hold ~150 μL of liquid and the surface tension caused by the Luer-lock was too great to permit an even passage of liquid under vacuum. Briefly, the Luer-lock was cut off of the Swinnex fitting inlet to maximize the opening. Next, the tip of the 15 mL tube was removed and the end of the tube subsequently finely sanded so that when inserted into the inlet and assembled with the outlet it would not come in contact with the filter membrane. The two pieces were bonded using a cyanoacrylate-type glue and allowed to cure for 24 hours. For filtration, the inlet/funnel was screwed onto the outlet portion of the Swinnex, which was connected to a vacuum source. This filtration apparatus is inexpensive (< $20 USD) and in combination with a manifold, allows for high throughput filtration.

### Enumeration of VLP using 13 mm Anodisc membranes

Our protocol for preparing virus slides using 13 mm Anodisc membranes is based on that of Ortmann and Suttle (2009), with modifications of the staining procedure. Back-staining is the standard protocol for Anodisc 25 membranes and involves placing the membrane sample side up onto a drop of stain, incubating, then removing excess stain by either wicking [14] or applying vacuum [12]. However, back-staining is technically challenging due to the small size and absence of a support ring on the 13 mm membranes. Thus, samples were pre-stained prior to filtration. The detailed protocol is as follows: i) A virus sample was brought up to a final volume of 900 μL using 0.02-μm filtered diluent (AN media or seawater). ii) 100 μL of SYBR Gold (25 ×, 0.02 μm filtered) was added to the sample and then incubated for 15 min in the dark. iii) A backing filter (0.2 μm, polyethersulfone, Pall Corporation, Port Washington, NY) was placed onto the screen of the Swinnex outlet and overlaid with sterile MilliQ water (~2 mL). Vacuum pressure (5 in Hg) was applied to pull the water through and stopped immediately so not to dry out the filter. iv) The backing filter was overlaid with MilliQ water (~2 mL) again and a 13 mm Anodisc placed on top of the water. v) The vacuum was then applied to pull the water through and sandwich the filters together. vi) With the vacuum still on, the modified Swinnex inlet (containing a gasket) was carefully screwed on and tightened with sufficient torque; excessive torque would crack the membrane and insufficient torque caused particles to be preferentially filtered towards the periphery of the membrane. vii) The sample was added to the center of the funnel. After all the liquid had visually disappeared, the vacuum was continued for an additional 30 seconds. viii) With the vacuum still on, the Swinnex inlet was carefully unscrewed, leaving the gasket and the two filters on the outlet. ix) The vacuum was cut and the three pieces (sandwiched filters and gasket) were removed as one and placed on Whatman (grade 4, qualitative) paper to dry for one min. x). Using forceps and a needle, the gasket was removed and the filters separated. xi) The Anodisc was mounted on a glass slide with anti-fade solution (50% glycerol, 50% PBS, 0.1% *p*-phenylenediamine). Filtration time was < 5 min per mL. Parallel samples were also prepared with a post-stain rinse, where 500 μL of 0.02-μm filtered media or seawater was added to the funnel and pulled through with the vacuum.

Enumeration was performed on a Leica DMRXA using filter cube L5 (excitation filter BP 480/40, suppression filter BP 527/30). For each slide, 20 fields and at least 200 particles were counted. To calculate the concentration of virus particles ml^-1^, the average number of particles per field was multiplied by the dilution factor and microscope conversion factor and then divided by the volume of sample filtered (in ml). The microscope conversion factor was calculated as the filterable area of the membrane divided by the area of each individual field. Variance in the filterable area using the meniscus loading method for the 25 mm Anodisc filters and the Swinnex filter holders for the 13 mm filters was 18.38 (± 0.115) and 9.61 (± 0.131), respectively.

### Comparison of VLP counts using Anodisc membranes and evaluation of staining methods

VLP concentrations were determined from three sample types with both Anodisc membranes: a viral lysate of a marine cyanobacterium, open ocean surface seawater and coastal surface seawater. Three replicate slides were prepared for each sample type and method. Previous studies have recommended a rinse step following staining of Anodisc 25 mm membranes when processing natural samples with high organic matter content (e.g. sediments, humic waters) to reduce background fluorescence [15]. Thus, we conducted a comparison of rinsing and no rinsing for both Anodisc membrane sizes across the three sample types. We also compared staining approaches (back- vs pre-) for the Anodisc 25 mm membranes. The cyanophage viral lysates gave indistinguishable VLP counts (ANOVA, *P *> 0.05) regardless of membrane diameter, staining and rinsing procedure. The two environmental samples showed variation among the methods tested that were due to the rinse step. Viral abundances determined using the two Anodisc membranes were significantly different (ANOVA, *P *< 0.05) when the post-rinse step was omitted. However, differences were not significant between the two membrane types when the post-rinse step was applied (ANOVA, P > 0.05) (Table 2). Replicate seawater samples had a higher coefficient of variation (5-30%) than phage lysates (5-10%). The higher variance amongst the replicate seawater samples is attributed to sample microheterogeneity. Microbial heterogeneity in natural aquatic samples is well known; bacteria and viruses have been shown to form aggregates or be in close association with organic particles [16,17].

**Table 2:** Comparison of back-staining and pre-staining of Anodisc membranes in VLP enumeration of three sample types

Sample	Filter^a^	Staining method	Rinse	VLP ^b^	CV ^c^
	Ano 25	Back	No	1.32 × 10^6 ^(0.08)	5.7
	Ano 25	Back	Yes	1.32 × 10^6 ^(0.10)	7.5
Cyanophage lysate	Ano 25	Pre	No	1.63 × 10^6 ^(0.07)	4.5
	Ano 25	Pre	Yes	1.54 × 10^6 ^(0.15)	9.6
	Ano 13	Pre	No	1.29 × 10^6 ^(0.13)	10.1
	Ano 13	Pre	Yes	1.26 × 10^6 ^(0.07)	5.8

	Ano 25	Back	No	9.59 × 10^5 ^(1.86)	19.4
	Ano 25	Back	Yes	1.66 × 10^5 ^(0.37)	22.5
Sargasso Sea water	Ano 25	Pre	No	7.50 × 10^5 ^(1.30)	17.3
	Ano 25	Pre	Yes	1.75 × 10^5 ^(0.17)	9.7
	Ano 13	Pre	No	5.93 × 10^5 ^(1.15)	19.3
	Ano 13	Pre	Yes	2.28 × 10^5 ^(0.54)	23.5

	Ano 25	Back	No	14.99 × 10^5 ^(0.45)	3.0
	Ano 25	Back	Yes	3.22 × 10^5 ^(1.06)	32.9
Southeastern US coastal waters	Ano 25	Pre	No	4.41 × 10^5 ^(0.62)	13.9
	Ano 25	Pre	Yes	3.28 × 10^5 ^(0.35)	10.7
	Ano 13	Pre	No	2.58 × 10^5 ^(0.35)	13.7
	Ano 13	Pre	Yes	2.75 × 10^5 ^(0.41)	14.9

^a ^Anodisc™ 25 mm (Ano 25) and 13 mm (Ano 13) membranes

^b ^Average VLP abundance from triplicate filters along with the standard deviation

^c ^The percent coefficient of variation from 3 replicate measures.

Discrepancies in VLP counts due to staining method and post-rinsing are most likely a reflection of differences in concentration and composition of viral communities (in terms of size and fluorescence) as well as organic material in the natural samples. For example, coastal environments and other highly productive systems typically contain a higher proportion of eukaryotic algae in the plankton then do oligotrophic systems, such as the open ocean [18]. Viruses that infect algae are routinely isolated and have been shown to be quite large in size (capsid, 100-220 nm) and contain large genomes [19,20]. A higher proportion of smaller, less fluorescent viruses in the open ocean could contribute to lower VLP counts after post-rinsing. The issue of including a post-rinse in the processing of natural samples for VLP enumeration is environment dependent and beyond the scope of this report, which is designed to illustrate the comparability of sample processing with the 13 mm and 25 mm Anodisc membranes.

### Analysis of Nuclepore membranes

**Figure 2:**
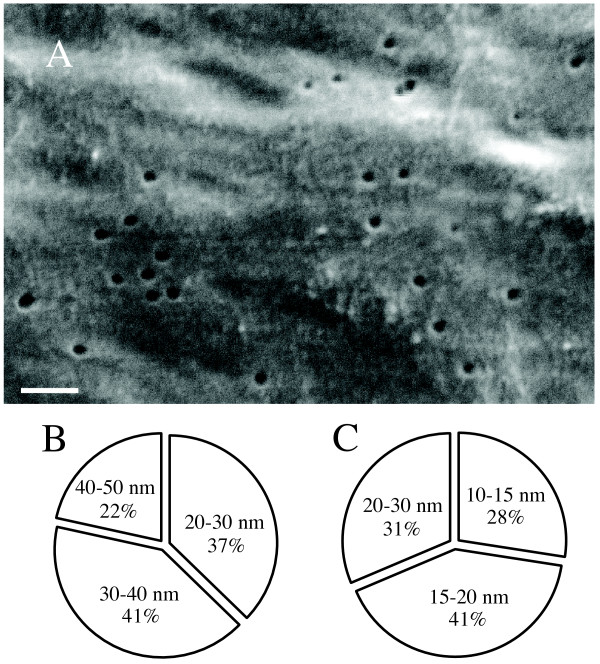
**Pore size distribution of untreated Nuclepore™ filters determined by SEM analysis**. (A) SEM image of Nuclepore™ 30 membrane. Scale bar is 200 nm. (B) Pore size range of Nuclepore 30 membrane. (C) Pore size range of Nuclepore 15 membrane.

The same samples described in the previous section were also processed using Nuclepore filters. Due to the low flow rate of Nuclepore membranes, filtering times have been traditionally quite long (> 1 hr). To maximize flow rates, existing protocols were modified. Specialized backing filters and filter holders were used and details are provided in the methods section. VLP enumeration from natural samples using Nuclepore membranes were generally an order of magnitude lower than parallel enumerations conducted using the Anodisc membranes (data not shown). Furthermore, analysis of Nuclepore filtrate subsequently passed through Anodisc membranes indicated VLP were passing through these membranes. Thus, Nuclepore membrane pore sizes were analyzed using scanning electron micrographs as described in the methods section. Pore sizes were consistent in membranes pre- and post-filtration. However, the pore sizes for Nuclepore 30 membranes were not uniform and ranged from 20 to 50 nm in size with the majority of pores being < 40 nm (78%)(Figure 2B); the Nuclepore 15 membranes were also not uniform and ranged from 10 to 30 nm in size with the majority of pores being < 20 nm (69%) (Figure 2C).

## Conclusions

Modifications of existing protocols allow the reliable use of Anodisc 13 membranes for enumeration of VLP using epifluorescence microscopy. In parallel studies, we found that Nuclepore filters (polycarbonate, 0.03 & 0.015 μm pore sizes) consistently yielded lower observable VLP. These low counts may be attributed to non-uniform pore sizes that were evident by scanning electron microscopy of these filters (Figure 2). However, more rigorous parallel comparisons of the Nuclepore and Anodisc membranes are necessary to determine this conclusively. Differences in VLP abundance estimates between Anodisc 13 and 25 membranes were evident with environmental samples if a post-rinse step was not included in sample processing. While rinsing of membranes gave the most consistent results across the two Anodisc membranes, it may result in loss of enumeration of VLP depending upon the environment from which the sample was derived. Given the heterogeneity of natural virus populations, individual investigators will need to consider the issue of applying a post-rinse on a case-by-case basis.

## Methods

### Sample collection and preparation

Viral lysate was made using cyanophage S-PWM1, which infects *Synechococcus *sp. WH7803 (aka DC2) [21]. The lysate was filtered through a 0.2-μm Durapore™ filter and stored at 4°C - this filtered material served as the lysate standard. Open ocean water samples were collected from the Sargasso Sea (May 28, 2005; 36.343° N, 51.315° W) and coastal water samples were collected off the coast of Georgia, USA (Nov 18, 2007; 31.372° N, 80.561° W). Multiple seawater aliquots (2 mL) were uniformly distributed, fixed in 0.5% glutaraldehyde and frozen at -80°C at the start of this study to ensure reproducibility.

### Enumeration of viruses using 25 mm Anodisc membranes

The protocol using 25 mm Anodisc membranes follows that published by Ortmann and Suttle (2009), with minor modifications. Briefly, filtration was performed on a Hoefer^® ^filtration manifold (Hoefer, Holliston, MA) without chimney weights. After the backing (0.45-μm pore-size cellulosics; MicroSep™, GE Water & Process Technologies, Trevose, PA) and the Anodisc filter were mounted on the filter stage with the vacuum on, the sample (final volume 1 mL) was applied to the top, forming a meniscus. The filter was back-stained by placement sample side up onto 100 μL of SYBR Gold stain (25 × concentration, Invitrogen, Carlsbad, CA) and incubated for 15 min followed by application of a vacuum to remove the stain. Samples were also prepared with a post-stain rinse of 850 μL of 0.02 μm filtered media or seawater. For direct comparison to the Anodisc 13 membranes, parallel samples were also pre-stained in a microcentrifuge tube prior to filtration. Filtration time using the above protocol was < 5 min per mL of sample.

### Determination of filterable area for Anodisc membranes

The filterable area of the Anodisc membranes was determined by passage of a cell culture of the naturally pigmented bacterium *Synechococcus *sp. WH7803 through them. Digital images were analyzed with Adobe^® ^Photoshop^® ^CS4 (Adobe Systems Incorporated, San Jose, CA) to calculate the area containing pigmented cells. The data reported is a range of the averages obtained from triplicate filters.

### Enumeration of viruses using Nuclepore membranes

As pre-stained black Nuclepore membranes with pore sizes of 15 and 30 nm are not commercially available, membranes were stained using 0.2% Irgalan Black (Acid black 107, Organic Dyestuffs Corporation, East Providence, RI) dissolved in 2% acetic acid as previously described [8], with the exceptions that staining time was reduced from 3 hours to 15 minutes and filters were used immediately. Polyester drain discs (Whatman), which are designed to improve flow rate and provide a flat surface to eliminate rupturing were used as backing filters. Filters were placed in 25 mm Swinnex filter holders for filtration and processed using the same reagents and solutions described for the Anodisc membranes. The filtration time required for the Nuclepore 15 and 30 membranes using the above protocol was < 60 min and < 10 min per mL, respectively.

### SEM imaging of Nuclepore membranes

To assess whether the filtration protocol could be damaging or altering membrane pore size, scanning electron micrographs of the Nuclepore membranes were taken before and after filtrating media (0.02 μM filtered AN) or seawater (0.02 μM filtered Sargasso Sea water) using a LEO 1525 field emission scanning electron microscope (Carl Zeiss Inc., Thornwood, NY, USA). Avoiding lateral stress, the membranes were cut, mounted on a stub and viewed. No coating was applied so as to not obscure the pores. At least 3 regions of each filter were viewed and at least 50 pores measured from each filter. Filtration did not appear to damage the filters or change pore size. Initial attempts at preparing the filters for SEM did suggest that lateral stress (excessive stretching or twisting) of the membranes could drastically increase pore size (data not shown).

### Statistical comparison of virus counts from the Anodisc membranes

The statistical software package SPSS was used to compare the VLP counts between the technical replicates (repeated-measures ANOVA, C.I. of 5%) and between the membrane types (2-tailed paired *t *test, C.I. of 5% or repeated-measures ANOVA, C.I. of 5%). Counts obtained from the individual fields of each slide were first evaluated using the Shapiro-Wilks test. Data sets that failed the Shapiro-Wilks test (having p-values < 0.05) were transformed using the Box-Cox transformation. The resulting transformed variables were consistent with a normal distribution. Mauchly's test of sphericity was performed and if the test was found to be significant (having p-values < 0.05) either the Huynh-Feldt (for epsilon values > 0.75) or the Greenhouse-Geisser (for epsilon values < 0.75) correction was applied.

## Competing interests

The authors declare that they have no competing interests.

## Authors' contributions

CRB developed the filtration procedures, coordinated the experimental design, performed the statistical analysis, and drafted the manuscript. SNL carried out the filtration of the samples and their microscopic enumeration. GRL participated in the experimental design, helped develop the filtration procedures, and helped to draft the manuscript. SWW participated in its design and coordination, and helped to draft the manuscript. AB participated in the design and coordination of the study, aided in the interpretation of the data, and helped to draft the manuscript. All authors read and approved the final manuscript.
